# A *de novo* variant of the *COL3A1* gene: causality of vascular Ehlers-Danlos syndrome

**DOI:** 10.1515/almed-2025-0172

**Published:** 2025-11-26

**Authors:** Estrella Gutiérrez Romero, Nuria Padilla Apuntate, Silvia Izquierdo Álvarez

**Affiliations:** Servicio de Bioquímica Clínica, Genética Molecular, Hospital Universitario Miguel Servet, Zaragoza, Spain

**Keywords:** *COL3A1*, frailty, ictus, vascular Ehlers-Danlos syndrome

## Abstract

**Objectives:**

To establish diagnosis of vascular Ehlers-Danlos syndrome (VEDS) through genetic testing. This syndrome is characterized by muscle and arterial ruptures and a tendency to easy brusing, thin skin with visible veins and acrogeric facial features that may be caused by pathogenic variants (PVs) in heterozygosis in the *COL3A1* gene.

**Case presentation:**

We present the case of a man who suffered a cerebrovascular accident. Following investigations, a *de novo* probably-pathogenic variant was identified in the *COL3A1* gene, which directed initial suspicion from Marfan syndrome to a final diagnosis of VEDS.

**Conclusions:**

The variant detected in the *COL3A1* gene may support causality in the clinical context of this patient and contribute to improving therapeutic management in similar cases.

## Introduction

Ehlers-Danlos syndrome (VEDS) is characterized by a higher risk of arterial and intestinal rupture, uterine rupture during pregnancy, and easy bruising, thin skin with visible veins and achrogeric facial features. This autosomal dominant condition is caused by a heterozygous mutation in the *COL3A1* gene.

## Clinical presentation

**Figure 1:**
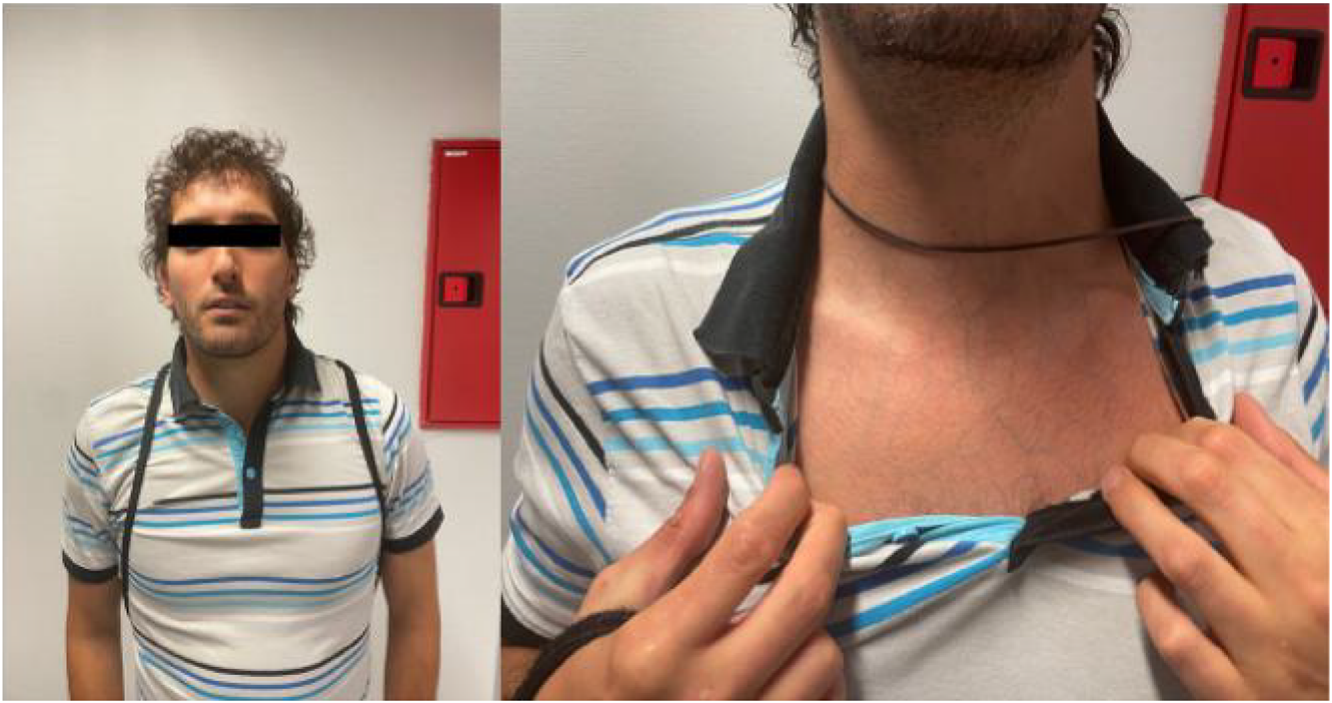
Acrogeric facial features: “beaked” nose, thin upper lip, prominent eyes, sunken cheeks. Visible veins, thin, translucent skin.

A 35 year-old male patient with a history of muscle frailty including multiple ruptures at the level of the biceps, pectoralis major muscle, and penis (three episodes). Patient phenotype: acrogenic facial traits, thin, translucent skin (Figure 1).

Family history; idiopathic death of his father following a bronchoscopy.

In January 2025, during a water polo match, he developed right-sided neck pain that radiated to the ipsilateral eye, along with dizziness and amaurosis. Subsequently, the patient experienced drowsiness, dysarthria and weakness in the left extremities and was transferred to a hospital.

The patient reported that in the days before, he had been working on building a house, frequently straining his neck and lifting heavy loads. The day of the episode, he felt right hemicranial discomfort, which he later identified as the same type of pain. He initially attributed it to the swimming cap and loosened it. After launching the ball, he noticed that he had lost vision in his right eye.

On examination, the patient was found to be hypotense (104/59 mmHg), drowsy, with moderate dysarthria, left homonymous hemianopia, right anisocoria with mild ptosis, severe left central facial palsy, left brachiocrural hemiplegia associated with hemianesthesia, and a National Institute of Health Stroke Scale score of 20.

Computerized tomography revealed right carotid dissection with a thrombus in the middle cerebral artery (MCA). Upon suspicion of right-sided acute ischemic stroke (total anterior circulation infarction) secondary to carotid dissection with a thrombus in M1 of the right MCA, intravenous fibrinolysis therapy was initiated. The patient was referred for a thrombectomy. However, in the absence of total vessel occlusion and presence of complete MCA recanalization, the procedure was interrupted due to the high risk for embolism.

Laboratory testing revealed slightly elevated levels of triglycerides and C-reactive protein (CRP) (Table 1).

**Table 1:** Laboratory test results for the patient.

Parameter	Value	Units	Reference range
Serum NT-proBNP	32	pg/mL	<103
Serum TSH	1.68	μUl/mL	0.38–5.33
CRP	1.25^a^	mg/dL	<0.50
Serum urea	30	mg/dL	17–43
Serum creatinine	0.78	mg/dL	0.67–1.17
Serum triglycerides	194^a^	mg/dL	30–175
Serum cholesterol	209	mg/dL	120–220
Total serum bilirubin, mg/dL	0.47	mg/dL	0.30–1.20
Total serum proteins	7	g/dL	6.60–8.30
GFR-CKD EPI	116.95	mL/min	≥90
Serum ALP	47	U/L	30–120
Serum GGT	36	U/L	<55
Serum GOT	19	U/L	<50

TSH, thyroid-stimulating hormone; CRP, C-reactive protein; GFR-CKD EPI, glomerular filtration rate-chronic kidney disease epidemiology collaboration; ALP, alkaline phosphatase; GGT, gamma-glutamyl transferase; GOT, glutamic oxaloacetic transaminase. ^a^Indicates red flag.

The patient had a benign 24-h progress and was discharged home. A blood sample was obtained for genetic testing including an exome panel for connective tissue disorders. Treatment with statins and adiro was initiated.

Subsequently, genetic testing using Next Generation Sequencing (NGS) with a targeted clinical exome was performed at LABGENETICS S.L. The analysis covered the coding regions of 138 genes (Table 2), as well as adjacent intronic regions (5pb) for the detection of single-nucleotide variants and copy number variations (CNVs) potentially associated with Marfan syndrome, hyperlaxity, aortic disease, Ehlers-Danlos syndrome and related disorders.

**Table 2:** Genes included in the targeted exome panel.

Genes associated with Marfan syndrome, joint hyperlaxity, aortic diseases, Ehlers Danlos syndrome and associated disorders (138 genes)
*ABCC6, ABCC9, ABL1, ACTA2, ADAMTS10, ADAMTS17, ADAMTS2, ADAMTSL2, ADAMTSL4, AEBP1, ALDH18A1, ATP6V0A2, ATP7A, B3GALT6, B3GAT3, B4GALT7, BGN, BMP1, BPNT2, BRAF, C1R, C1S, CANT1, CBS, CHST14, CHST3, COL11A1, COL11A2, COL12A1, COL1A1, COL1A2, COL2A1, COL3A1, COL4A5, COL5A1, COL5A2, COL6A1, COL6A2, COL6A3, COL9A1, COL9A2, COL9A3, CRTAP, CST3, DLG4, DSE, EFEMP2, ELN, ENPP1, EXOC6B, FBLN5, FBN1, FBN2, FGD1, FGFR3, FKBP10, FKBP14, FLNA, FLNB, FMR1, FOXE3, GAA, GALNS, GATA5, GGCX, GORAB, HCN4, HRAS, IFITM5, KCNJ8, KDM6A, KIF22, KMT2D, KRAS, LOX, LTBP2, LTBP3, LTBP4, LZTR1, LZTS1, MAP2K1, MAP2K2, MAT2A, MED12, MFAP5, MSTN, MYH11, MYH7. MYLK, MYLK2, NKX2-5, NOTCH1, NOTCH3, NRAS, P3H1, PLOD1, PLOD2, PLOD3, PLP1, PPIB, PRDM5, PRKG1, PTPN11, PYCR1, RAF1, RIN2, RIT1, ROBO3, RYR1, SERPINH1, SKI, SLC26A2, SLC2A10, SLC39A13, SMAD2, SMAD3, SMAD4, TTN, SMS, SOS1, SOS2, SP7, SSPN, TAB2, TGFB2, TGFB3, TGFBR1, TGFBR2, TNFRSF1A, TNXB, TRPS1, TTN, TYMP, UPF3B, VCAN, XYLT1, ZDHHC9, ZNF469*

Exome Panel v2.5 (Illumina) was used for library preparation and capture probe-based enrichment, targeting the coding regions (splicing ± 5pb regions) of 19,396 genes and achieving an on-target rate of over 97 % at 10× depth for detection of single-nucleotide variants (SNVs and Indels) and CNVs (large deletions/duplications). Sequencing using Illumina NextSeq^®^2000 (Blackhills Diagnostic Resources (BDR), Zaragoza, Spain). Bioinformatics analysis and alignment of the sequences obtained against the reference genoma hg19 using the Dragen v4.3 software package (Illumina). Variant analysis and interpretation were performed using Illumina Emedgene, ACMG score, Alamut^®^ Visual 1.9, gnomAD browser, Varsome and ClinVar. Validation of the informed variants was carried out by Sanger sequencing.

Sanger sequencing identified a probably-pathogenic variant (VPP), in the *COL3A1 *gene, c.1977+1G>C, p.(?), which could involve the donor splice site (5′-donor splice site) of intron 28 of the *COL3A1 *gene. This variant could introduce a failure in protein splicing resulting in an aberrant splicing in mRNA of the protein encoded by this gene, thereby rendering it nonfunctional.

This variant had never been described before as a pathogenic variant (PV) associated with the development of any disease, nor is included in any of the databases searched (LOVD, ClinVar, dbSNP, gnomAD). Therefore, it was an unknown variant.

In accordance with the American College of Medical Genetics (ACMG) criteria, the variant was classified as a PPV (PVS1, PM2). Since the presence of PVs in the *COL3A1* gene are associated with vascular Ehlers-Danlos syndrome (OMIM#130050), its presence in heterozygosis would support diagnosis of this disorder and the causality of symptoms [1].

No PVs or PPVs were identified in any of the 81 genes that the ACMG (v3.2) recommends testing in the presence of secondary findings [2].

In the light that Ehlers-Danlos syndrome (EDS) caused by a PV in the *COL3A1* gene has an AD inheritance pattern, genetic testing for this variant was recommended in the patient’s first-degree relatives.

## Discussion

Initial suspected diagnosis relies on a family or personal history of arterial dissections, ruptures or aneurysms or pregnancy complications at an early age. As many as 50 % of patients may harbor *de novo* mutations [3].

VEDS accounts for 4–5 % of cases of EDS. This syndrome is associated with a high rate of fatal complications related to the cardiovascular and gastrointestinal systems, including cerebrovascular hemorrhage or intestinal rupture [4].

Although muscle ruptures are considered minor diagnostic criteria for VEDS, muscle-skeletal involvement alone is not frequent in this disease. In the absence of a relevant family history, diagnosing VEDS in patients with a history of muscle ruptures alone is challenging, even when these are recurrent [5].

In the presence of this phenotype, Marfan syndrome and other collagenopathies should be considered in the differential diagnosis, as they share clinical features. Indeed, after Marfan syndrome has been ruled out via the Marfan systemic scale or genetic testing for *FBN1*, clinicians should consider any form – possibly atypical – of collagenopathy [6].

VEDS diagnosis requires molecular confirmation through the identification of a PV in one allele of the *COL3A1* gene, in order to distinguish this syndrome from other clinically similar conditions [7].

Diagnostic criteria for VEDS should be considered in individuals meeting either a major criterium or several minor criteria. Major criteria: arterial aneurysms, dissections or ruptures, intestinal rupture, uterine rupture during pregnancy and/or a family history of VEDS. Minor criteria: pneumothorax, easy bruising, thin translucent skin, clubfoot, acrogeric facial features, muscle or tendon rupture, varicose veins at a young age, and subluxations, to name a few [8].

The broad clinical spectrum is explained by allelic heterogeneity. Common clinical manifestations include: spontaneous pneumothorax (12 %), coronary artery dissection resulting in myocardial infarction, spontaneous rupture of the sigmoid colon, superficial venous insufficiency (37 %), carotid cavernous fistula, ocular protrusion and keratoconus, gingival fragility (bleeding after teeth brushing or flossing), as well as temporomandibular joint hypermobility (repeated subluxations) and renal artery dissection which may lead to decreased renal blood flow, loss of renal parenchyma and renal hypertension [9].

Over 1,500 variants of the *COL3A1* gene have been reported, each associated with a pathogenic phenotype. Most of the PVs identified result in substitutions of one aminoacid to glycines in the Gly-XY repeat sequence of the triple helical domain of the type III procollagen molecule [10].

Mean survival is 51 years, although it varies according to the type of PV nature, with patients carrying a null allele exhibiting a longer life expectancy [11].

The primary goal of medical intervention is to maintain blood pressure within a normal or low range and prevent blood pressure peaks to minimize the risk for arterial dissection or rupture. Pharmacotherapeutic management includes diuretics, β-blockers, angiotensin processing blockers, and other antihypertensive agents [12].

## Conclusions

This is the first case report describing a *de novo* PPV in the *COL3A1* gene as a cause of VEDS. Proper diagnosis relies on careful evaluation of clinical features and, when indicated, genetic testing, whose results are pivotal for defining treatment and prognosis. Early management remains key to reducing disease burden and symptom severity. The laboratory, along with genetic testing, played a major role in the diagnosis of this case, enabling access of the patient and his direct relatives to genetic and reproductive counseling.

## Supplementary Material

Supplementary Material
